# Hollow Microneedles on a Paper Fabricated by Standard Photolithography for the Screening Test of Prediabetes

**DOI:** 10.3390/s22114253

**Published:** 2022-06-02

**Authors:** Tianwei Wu, Xueqiu You, Zhong Chen

**Affiliations:** 1 Department of Electronic Science, Fujian Provincial Key Laboratory of Plasma and Magnetic Resonance, State Key Laboratory of Physical Chemistry of Solid Surfaces, Xiamen University, Xiamen 361005, China; 34320191150168@stu.xmu.edu.cn; 2 School of Information Engineering, Jimei University, Xiamen 361021, China

**Keywords:** photolithography, hollow microneedle, ISF, paper sensor

## Abstract

Microneedle (MN) is a novel technique of the biomedical engineering field because of its ability to evaluate bioinformation via minimal invasion. One of the urgent requirements for ground-breaking health care monitoring is persistent monitoring. Hollow microneedles are extremely attractive to extract skin interstitial fluid (ISF) for analysis, which makes them perfect for sensing biomarkers and facilitating diagnosis. Nevertheless, its intricate fabrication process has hampered its extensive application. The present research demonstrates an easy one-step preparation approach for hollow MNs on the foundation of the refraction index variations of polyethylene glycol diacrylate (PEGDA) in the process of photopolymerization. The fabricated hollow microneedle exhibited ideal mechanical characteristics to penetrate the skin. Hydrodynamic simulations showed that the liquid was risen in a hollow microneedle by capillary force. Furthermore, a paper-based glucose sensor was integrated with the hollow microneedle. We also observed that the MN array smoothly extracted ISF in vitro and in vivo by capillary action. The outcomes displayed the applicability of the MN patch to persistent blood glucose (GLU) monitoring, diagnosis-related tests for patients and pre-diabetic individuals.

## 1. Introduction

Diabetes often poses a serious health risk to patients. According to the report of the World Health Organization (WHO), there were an estimate of 422 million diabetic individuals in 2014 [1], and the number will increase to 592 million by 2035 [2]. For diabetic patients, a continuous blood glucose monitoring system enables precise and effective measurement of rapid changes in blood glucose levels for effective treatment [3,4]. As a rich sample of biological information, blood is a major source of samples for blood glucose testing. However, blood collection is often painful for the patient and requires specialists to reduce the potential risk of infection [5]. Skin interstitial fluid (ISF) is filtered by capillaries and possesses the same rich biomarkers as blood, with approximately 80% of proteins in serum contained in it as well, and ISF is an effective alternative source to blood [6,7,8]. ISF can be sampled using techniques such as blistering and capillary ultrafiltration, methods which are intricate and harbor the risk of infection [9]. Hence, a more convenient, easy-to-use method of extracting samples for ISF is needed [10].

Microneedles are a new painless physical facilitation technique that can be used for transdermal administration and bio-sensing [10,11]. Microneedles are generally less than 1 mm in length and can penetrate the epidermis without damaging nerves, and its utilization does not require professional guidance. There are generally four types of microneedles: hollow microneedles, solid microneedles, porous microneedles and hydrogel microneedles. Solid microneedles are usually made of conductive materials or coated with conductive materials and can be used as sensing electrodes for bio-signal monitoring [12,13,14]. Hydrogel microneedles generally absorb ISF via the ability to swell [15,16]. Although the process of making hydrogel microneedles is simple, an additional step is required to separate the extracted ISF before analysis. Porous microneedles with interconnected pores allow the extraction of ISF by capillary action, whereas it takes more than 2 h to extract sufficient ISF for analysis [17]. The long extra sampling time can bring an unpleasant usage experience for the patient. As the body’s metabolites change over time, a longer sampling time can reduce the accuracy of metabolite analysis. Therefore, equipment with shorter sampling time has priority in terms of biological monitoring. The hollow microneedle consists of a cavity of a certain length and an oblique opening, which can be used for drug delivery and for the extraction of ISF for bio-analysis [18,19,20]. It presents better drug delivery capabilities compared to solid, soluble microneedles [21]. Hollow microneedles are generally made of glass, metal, silicon, ceramic and require the use of Micro-Electro-Mechanical System (MEMS). Although hollow microneedles made of silicon exhibit ideal physical properties, the high material cost and complicated fabrication process constitute their disadvantages [19,22]. In contrast, the use of diffraction lithography and a special mask design in this paper allows hollow microneedles to be fabricated in a single pass [23,24]. The mixture of polyethylene glycol diacrylate (PEG-DA) and photoinitiator can be considered a negative photoresist. PEG-DA can be cured under UV light irradiation [25,26]. PEG-DA is used due to its satisfactory biocompatibility and sufficient mechanical properties to penetrate the skin after curing [27,28,29].

The use of paper substrates, which merely requires a small amount of samples, as the interest regarding microfluidic analysis equipment has also arisen [30,31]. The advantage of the paper substrate is the capillary action of the pore-rich structure, which allows the sample to reach the entire analytical area [32]. The pore-rich architecture also offers a great superficial area as an ideal matrix medium for immobilizing macro-molecules, such as enzymes. Therefore, the use of paper substrates as analytical equipment is a fairly effective solution.

In this paper, hollow microneedles are fabricated by photolithography and the combination with glucose paper for ISF sampling and analysis, thereby forming a colorimetric glucose paper sensor. When the integrated microneedle patch pierces the skin, the ISF reaches the glucose paper sensor via the hollow microneedle through capillary action. The concentration of glucose can be measured by the change in the color of the test paper. The extraction aspects of ISF were evaluated by in vitro experiments with microneedle patches and in vivo experiments in rats. A sufficient sample was extracted in about 5 min to induce a color change in paper test strips. Therefore, the microneedle patch can be used as an effective screening test device for pre-diabetic patients.

## 2. Materials and Methods

### 2.1. Materials

All chemicals are commercially available and used in the required manner. We purchased PEG-DA with number average molecular weight, Mn = 250, and 2-hydroxy-2 -methylpropio-phenone, a water-soluble photo-initiator (Sigma-Aldrich, St. Louis, MO, USA). Polydimethylsiloxane (PDMS) was purchased from Dow Corning, Midland, MI, USA. Glucose test strips were purchased from Urit, China. The cellulose powder was purchased from RHAWN Chemical, China. The photomask was purchased from Micro and Nano Research Laboratory from Shenzhen, China.

### 2.2. Fabrication of the Glucose MN Sensor

**Figure 1:**
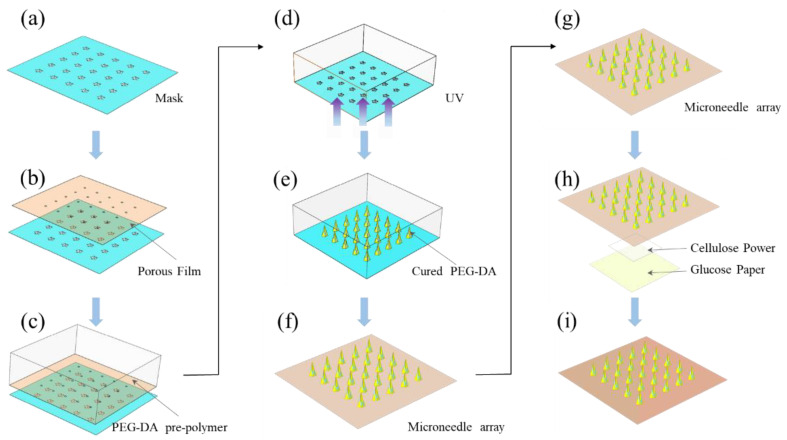
Fabrication of MN patches. (**a**) A PEG-DA film with microholes was fabricated. (**b**) The PEG-DA film was placed by aligning with the photomask. (**c**) PEG-DA solution was combined with the film and photomask. (**d**–**f**) UV exposure through the photo mask produced MN arrays on PEG-DA films. (**g**–**i**) MN array was integrated with the glucose test paper, and cellulose powder was added in the middle.

The complete glucose MN sensor consisted of two parts: the hollow microneedles based on a micro-hole PEG-DA film and the glucose-reactive test paper. A pore-rich PEG-DA film with a 160 μm diameter pore size and a thickness of 100 μm was prepared as a substrate. The fast and high flux through-hole film preparation technology was used to fabricate the PEG-DA film with microholes [33]. Then, the porous film was aligned with the opaque circle on the mask pattern. The photomask was made of soda glass plated with a thin chrome. The thickness was 1.6 mm. The porous film aligned with the mask was placed on top of the PDMS recess containing the UV-curable solution (Figure 1a–c). The solution was a mixture of the 99.5 weight% of PEG-DA and 0.5 weight% of 2-hydroxy-2-methylpropiophenone. Microneedles were produced using UV light to pass through the mask pattern (Figure 1d–f). Ethanol and deionized water were used to rinse the cured hollow MN array several times to wipe off uncured residual solution. Subsequently, it was dried with a nitrogen gun. The prepared glucose paper was integrated with hollow MN by double-sided adhesive and the middle of both layers was filled with cellulose powder to form a complete glucose MN sensor (Figure 1h,i) [34]. Once the hollow MN array penetrated the skin, the ISF reaches the response area of the top glucose sensor due to capillary action; thus, the glucose concentration was identified.

### 2.3. In Vivo MN Insertion Test Protocol

#### 2.3.1. Scanning Electron Microscopy (SEM)

The characteristics of hollow MNs were assessed via SEM images. The polymerized PEG-DA did not have electrical conductivity and required gold plating to obtain clear SEM images.

#### 2.3.2. Force Displacement Test

The mechanical strength of the hollow microneedles was evaluated using a universal testing machine. The microneedle array was placed facing upwards and the force gauge was moved downwards at 1.2 mm/min to obtain a displacement force relationship curve.

#### 2.3.3. Image Analysis

Images of rats were taken with a Canon digital camera. Optical images of the paper sensor before and after the insertion of the agar gel were obtained by a scanner. The resulting optical image was imported into the OPENCV module for fitting to obtain the size of the reaction zone. The RGB average value of each pixel in each reaction area was identified by Python. The difference between the RGB average of the paper sensor after each response and the non-response was used as the brightness value. This brightness value provided a measure of the change in color intensity before and after the glucose determination reaction.

### 2.4. In Vivo and Vitro MN Insertion Test Protocol

The insertion experiments were performed on rats. During the experiment, rats were anaesthetized by the inhalation of isoflurane to reduce pain. First, SD rats was placed in a box and anesthetized with 3% isoflurane. They were then transferred to a heating plate and the inhaler was continuously administered with 1% isoflurane. The skin of the rat was exposed by removing the hair using a razor. For the ISF extraction efficacy of microneedles, agar gels of different concentrations were prepared, and microneedle arrays were inserted for testing. The top of the gel was covered with a layer of aluminum (Al) foil.

Animal care and experimental procedures used in the current study were approved by the Animal Care and Use committee of Xiamen University (Permit Number: XMULAC20210083). The study was carried out adhering to guidelines provided by National Institutes of Health for the Care and Use of Laboratory Animals and all efforts were made to minimize suffering of animals.

## 3. Results and Discussion

### 3.1. Principle of Hollow Microneedle Design

**Figure 2:**
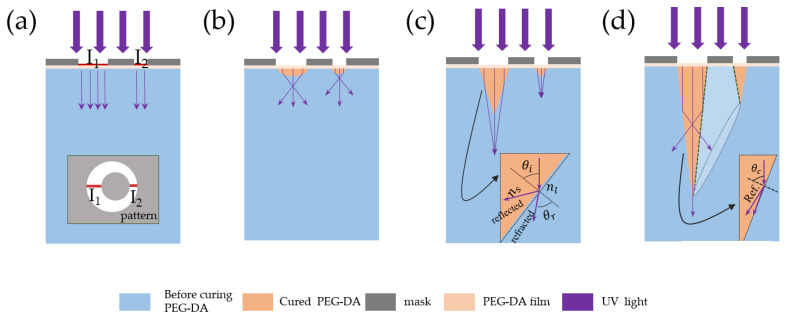
Principle of lithography on the foundation of variable refraction index PEG-DA. UV refracted at the interfacial region of the uncured and photocured PEG-DA. (**a**–**d**) The formation process of hollow microneedles.

As presented by Figure 2a, the light-transmitting parts of the pattern were asymmetrical. I_1_ and I_2_ are the maximum and minimum values of the aperture diameter, respectively. When UV light passed through the pattern on the mask into the PEG-DA monomer solution, the monomers near the pattern would be polymerized to form a lens-shaped morphology, as presented by Figure 2b. The cured lens-shape had a greater refraction index in contrast to the uncured area; thus, the UV light was focused, and a cone-shaped curing zone was formed. Since polymerized PEG-DA has a greater refraction index in contrast to its monomer, UV light was refracted at the interfacial region between the both materials with a refractive angle:

(1) $$\theta_{r}=sin^{-1}(n_{s}sin\theta_{i}/n_{l})$$

where $n_{l}$ and $n_{s}$ denote the refraction indexes of the uncured polymer and photocured polymer, respectively. As the difference in the refraction indexes between cured and uncured PEG-DA was 0.04, the reflected light could be neglected in the cone-growing phase (Figure 2c). The light refraction allowed the conical zone to grow persistently. The cone angle continuously became steep and reached a critical angle:

(2) $$\theta_{r}=\sin^{-1}(n_{s}/n_{l})$$

The UV light propagation became a total internal reflection, leaving only reflected light at the photocured/non-cured interface. If the exposure was stopped at the critical angle, it could obtain a single-step conical structure. The different light transmission widths on the pattern formed a single-step conic architecture with different heights. The pattern was a circular structure forming a hollow structure depicted by dashed lines, as show in Figure 2d Owing to the properties of the light ray focusing deriving from the different refraction indexes of the materials producing 3D architectures without any additional condition, this phenomenon is named as self-focusing photolithography [35].

### 3.2. Characterization of PEG-DA

**Figure 3:**
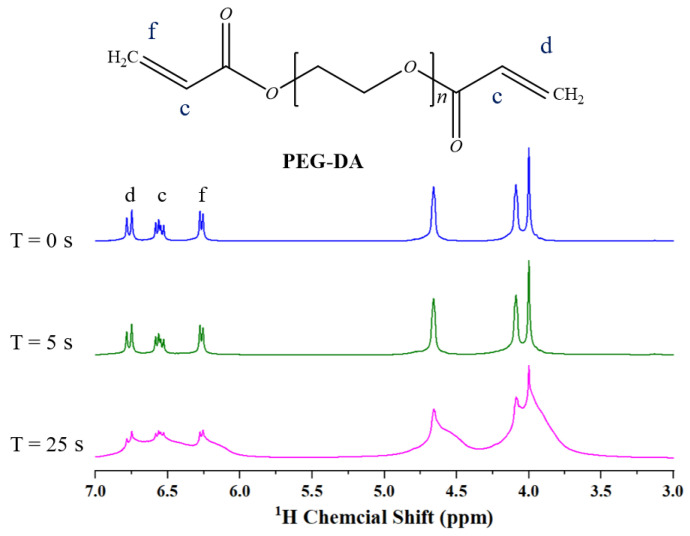
^1^H NMR spectrum of PEG-DA. c, d, f is the location of the hydrogen bond on PEGDA.

Due to its innocuous, good biocompatibility, low immunogenicity and ease of use, the PEG-DA is utilized extensively in the biomedical area. PEG-DA hydrogels have a viscoelastic, three-dimensional network structure. The acrylate double bond at the end of the PEG-DA molecule chain can be broken under UV irradiation and join with other PEG-DA molecules to form cross-linking points and networks. During photo-polymerization, the double bond is fully converted to form a polymeric network. The ^1^H NMR was utilized to verify the photopolymerization level of the PEG-DA monomer, as shown in Figure 3 A total of 50 µL solvent mixture was placed in the NMR tube. The tube was exposed at various times with an intensity of 10 mW/cm^2^ and a wavelength of 365 nm. The characteristic peaks of the acrylate double bonds became gradually weaker as the light exposure time increased. This indicates that the proportion of acrylate double bonds decreases as the degree of photopolymerization progresses. Eventually, the PEG-DA solvent was largely converted to a solid state.

### 3.3. Characterization of Hollow Microneedles

The sizes of the catheter opening, needle height, and tip profile are the important features of hollow microneedles, which determine their functions and mechanical stability. In the used preparation approach, those features were predominantly influenced by these aspects: diameters of inner and outer patterns, offset distance, UV strength, and total UV exposure time.

**Figure 4:**
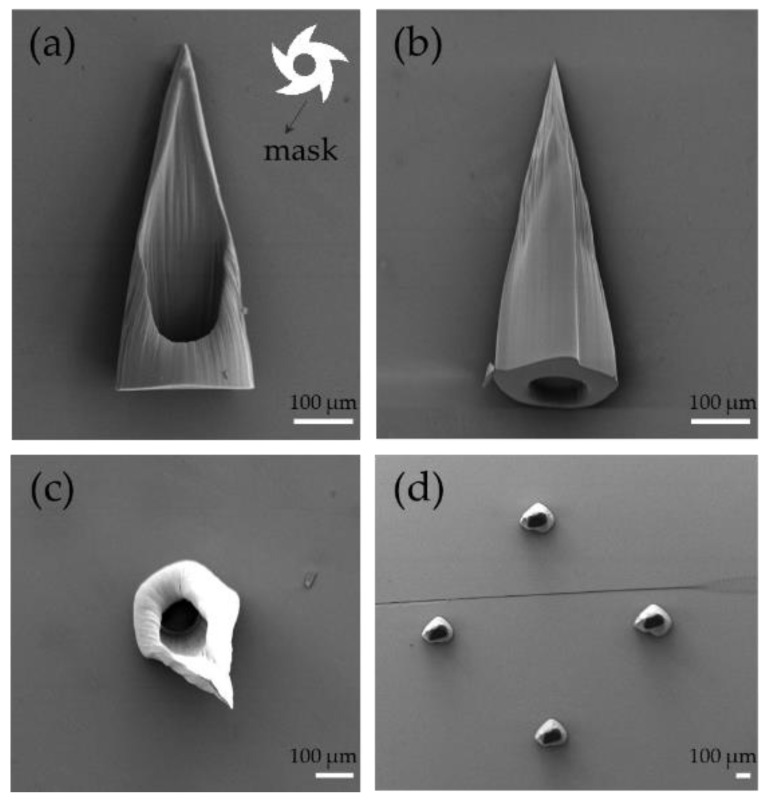
SEM image: (**a**–**c**) Front, back and top views of the hollow microneedle. The white part shows the detailed pattern of the photomask. (**d**) Microneedle array.

The hollow microneedle was obtained after the exposure. A side-slanted hole similar to a hypodermic syringe is shown in Figure 4a. The needle height was 600 μm. The tip of the microneedle was so sharp that it effectively penetrated the skin. The hollow channel enabled the efficient extraction of ISF. The outer multi-blade structure allowed the microneedle to create a blade structure from the base of the microneedle to the tip, which allowed the microneedle to penetrate the skin more effectively, as shown in Figure 4b. The use of photolithographic polymerization also enabled the bulk generation of microneedles into arrays. Figure 4d presents the outcomes of the fabrication of hollow MN arrays via patterned arrays.

**Figure 5:**
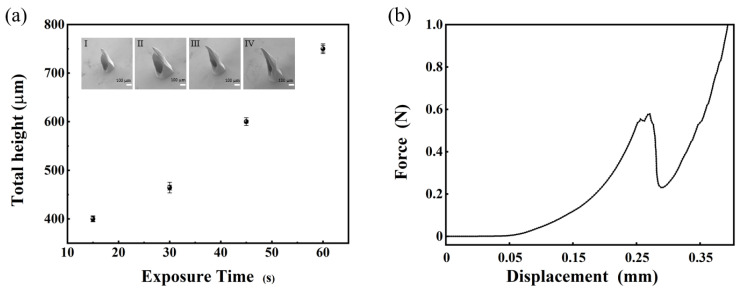
(**a**) Microneedle length and SEMs image at different exposure times. (**b**) The fracture force per needle of the exposure duration of 45 s.

The mask pattern of the microneedle was asymmetrical, as shown in Figure 4a. The outer diameter was a transparent multi-blade structure, internally tangent to a circle of 550 μm in diameter. The diameter of the opaque circle in the middle is 160 μm with 20 μm of offset distance of the inner pattern from the center. In order to optimize the design of the microneedle array, the length of the microneedles and the size of the catheter opening were obtained by changing exposure time from 15 s to 60 s with a UV intensity of 10 mW/cm^2^. The overall trend is shown in Figure 5a. With the increase in time, the height of microneedles also increased. When the exposure time exceeded 40 s, the opening of the hollow microneedle began to close, and the hollow channel could not be formed, as show in Figure 5a IV. The final selection of the hollow microneedle array was produced by an exposure duration of 45 s. When the light intensity was 30 mW/cm^2^, the result is shown in Figure S1d We also adjusted the deflection distance from 0 µm to 35 µm. The result is shown in Figure S1a–c Although light intensity and offset distance affect hollow microneedles, the limited data available do not allow a specific link to be obtained. Additional experiments and work may be required.

To assess the mechanical properties of the hollow MN arrays, compression experiments were carried out. An array of 13 × 13 microneedles was fixed to the slide with a double-sided tape and did not come off during the test. The relationship between displacement and force was used to assess its mechanical strength. The total peak force was compared to the number of microneedles to obtain the peak force of a single microneedle. Figure 5b presents the test outcomes of the single microneedle. The fracture force per needle was 0.6 N, which allowed the effective penetration of the skin.

### 3.4. Hydrodynamic Simulation of Extraction ISF

**Figure 6:**
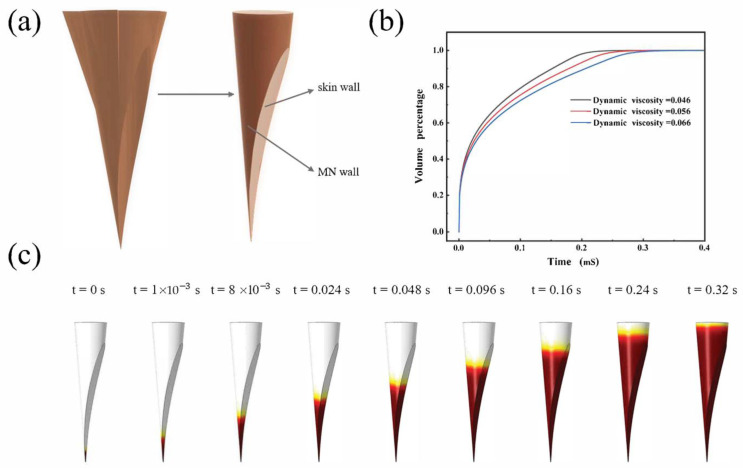
(**a**) Three-dimensional structure of hollow microneedles and formation of hollow channels by the MN wall and skin tissue. (**b**) Rising velocity of droplets with different kinetic viscosities; 0.056 is the standard value for ISF. (**c**) Extraction of ISF time-lapse simulation results, initially moving faster and then slower through the closed channel.

In order to reveal the hydrodynamic behavior of the hollow microneedle-extracted ISF, hydrodynamic simulations were carried out using the two-phase flow module in COMSOL Multiphysics. Fluid dynamics simulations showed that ISF acquisition occurred in an ultra-fast way with the help of capillary forces induced by the hollow microchannel structure of the hollow microneedles. Figure 6a presents a schematic diagram of the microchannels formed by the MN posterior to the skin penetration. When the hollow microneedle penetrated the skin, the oblique incision at the tip of the microneedle and the exposed side of the incised tissue formed a narrow lower and wide upper channel. By capillary action, the ISF could pass through this channel and pass through the micropores in the substrate to the paper sensor above. Figure 6c shows the process and speed of the ISF rise in the microchannel. Due to the narrow bottom and top wide microchannel structure, the rising speed of ISF gradually slowed down (Movie S1). The effect of dynamic viscosity on the process of MN extraction of tissue fluid was also investigated. As shown in Figure 6b, the fluid rises more slowly as the value of the dynamic viscosity becomes higher. However, the overall speed difference is not very large, indicating that the microchannel is relatively stable and able to cope with different situations.

### 3.5. Evaluation of Glucose MN Sensor

In order to evaluate the effectiveness of the glucose microneedle sensor, a GLU test was performed, which is one of the most extensively utilized biochemic analysis method. The GLU solution harvested by the microneedle reached to the top reaction tier, in which the oxidation of glucose occurred in the presence of the test paper. The test color varied into another color, which was determined by the GLU level. The powdered cellulose was used as a steady channel between the test paper and the MN layer. As the paper absorbed the sample and became wet, the hollow microneedle array, cellulose powder, and paper formed a connected and stable fluid channel. If the cellulose powder was not filled in, the color of the top layer of the paper would not be changed.

**Figure 7:**
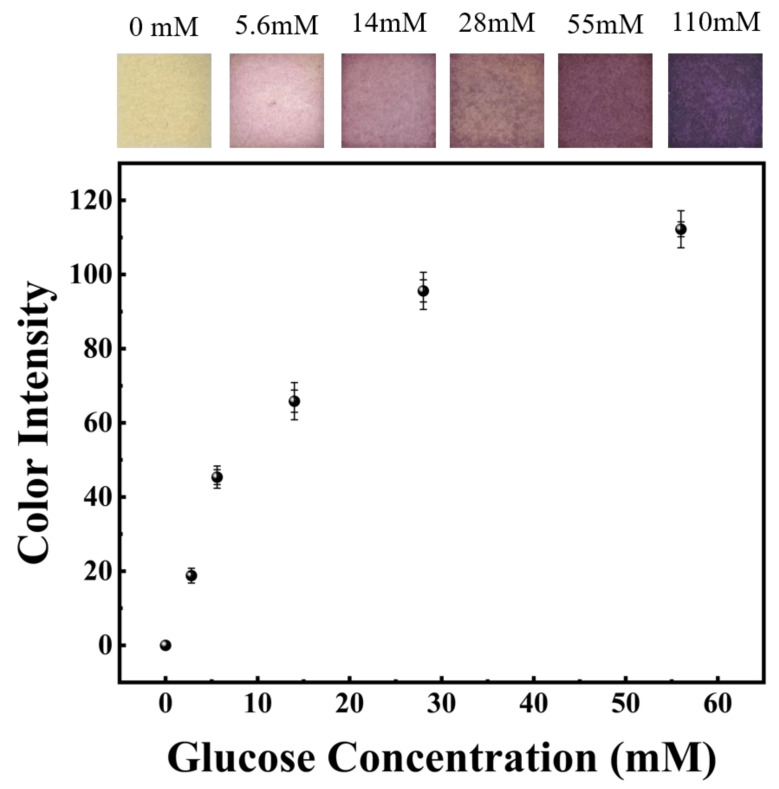
Scanned images of the reaction zone of the paper sensor with different glucose concentrations and the average color intensity of the reaction zone.

To test the performance of the extracted ISF of the glucose sensor, hollow microneedles were inserted into the agarose gel containing glucose. The top of the gel was covered with a layer of Al foil. Due to capillary forces, the extracted glucose solvent passed through the hollow channels of the hollow microneedles and reached the test paper tier. The test reagent on the test paper tier reacted with GLU, which was oxidized by glucose oxidase to produce glucose acid and H_2_O_2_. After that, under the influence of H_2_O_2_, the color of the test paper varied from yellow to pink. The glucose concentration was indicated by the color of the testing paper after the reaction. The scan picture and color strength of the reactive process were measured after the insertion of agarose gel at various glucose concentrations for 2 min, as shown in Figure 7 The outcomes showed a clear color variation owing to differences in GLU levels and was visually distinguishable. Starting with a light pink with a concentration of 5.6 mM, the color gradually varied to a deep purple and the color intensity increased rapidly.

**Figure 8:**
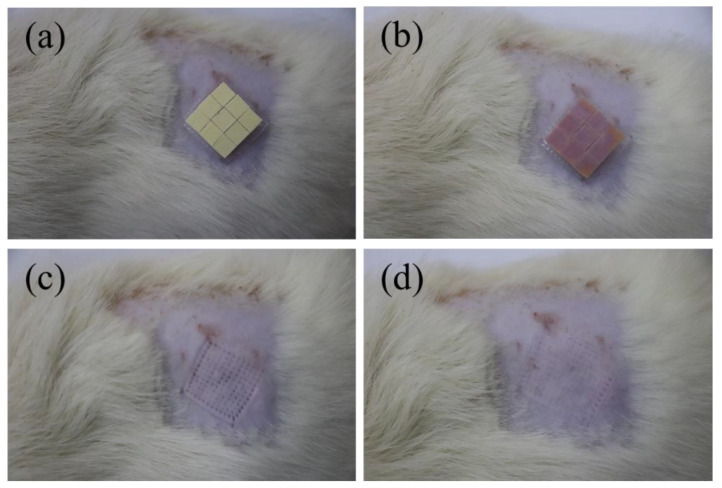
(**a**,**b**) The rats were shaved on the backs to expose their skin, and the microneedle patch was pressed in with the finger and the color varied after 4 min. (**c**) The microneedle patch was removed to produce the microholes. (**d**) After 30 min, the skin of the rats returned to the original state before the microneedle patch insertion.

To evaluate the ISF extraction performance of manufactured microneedle arrays, animal experiments were conducted using rats for insertion tests. The rats were anesthetized and the skin was exposed by shaving the back areas of the rats using a razor, as shown in Figure 8a. Then, the microneedles were fitted to the bare skin and then inserted into the rat using fingers. After approximately 4 min, the color of the paper on top of the sensor varied, indicating that the microneedle array successfully penetrated the skin of the mouse and the ISF entered the top of the sensor through a hollow microneedle and reacted with the test strip. This also meant that the measurement of blood glucose concentrations in rats was successfully completed. The comparison with the different concentrations of colorimetric cards obtained in previous agar gel experiments indicated that the blood glucose concentration in rats was around 5.6 mM, which was consistent with that of normal rats. When the MN patch was removed, microholes corresponding to the number of microneedles could be seen on the backs of the rats. As shown in Figure S2, a comparison of the hollow microneedles before and after insertion into the skin of the rats shows that only the tip of the needle is partially worn, which is consistent with the mechanical test results. After about 30 min, the small holes largely disappeared. MN patches did not cause harms to rats. Thus, the glucose concentration in ISF can be persistently measured by combining hollow MN arrays with glucose dry chemical reaction strips. The integrated MN patch can be used as a pre-tester to check one’s own blood glucose concentration in real time to determine whether a pre-diabetic patient needs to seek an accurate diagnosis. Furthermore, as the integrated MN patch has successfully demonstrated its usability with paper-based enzyme sensors, it is expected to provide an effective platform for diagnostic devices utilizing a variety of biosensors.

## 4. Conclusions

In this paper, a microneedle sensor was fabricated, which could successfully puncture the skin of rats to extract ISF and facilitate the rapid analysis of the absorbed ISF. Integrated microneedle sensors are simple, cost-effective and do not require additional intricate fabrication processes. It is a combination of hollow microneedles and glucose test strips. Under the action of capillary force, the sample absorbed by the microneedle flowed to the test strip through a vertical flow channel. The color of the test strip varied in correspondence to the concentration of glucose. The hollow microneedles were obtained by the photolithographic polymerization of PEG-DA by UV light through an unpaired mask pattern. By adjusting the exposure time and the offset distance of the opaque circle at the center of the mask pattern, microneedles with the right geometric structure and mechanical properties were obtained.

The microneedle sensor is easy to carry and can be easily applied by simply pressing in with a finger. It can measure glucose concentration and is suitable for real-time blood glucose monitoring in daily life. Hollow microneedles can be combined with other kinds of paper-based sensors, which makes them promising for the identification of various other biomarkers.

## Supplementary Materials

The following supporting information can be downloaded at: https://www.mdpi.com/article/10.3390/s22114253/s1, Simulation of liquid in a single hollow microneedle (Movie S1). Figure S1: (a–c) Optical images of different offset distances. (d) The microneedle heights for different UV intensity; Figure S2: The optical image before and after the penetration.

## References

[1] Zimmet P., Alberti K.G., Magliano D.J., Bennett P.H. (2016). Diabetes mellitus statistics on prevalence and mortality: Facts and fallacies. Nat. Rev. Endocrinol..

[2] Mo R., Jiang T., Di J., Tai W., Gu Z. (2014). Emerging micro- and nanotechnology based synthetic approaches for insulin delivery. Chem. Soc. Rev..

[3] Petrie J.R., Peters A.L., Bergenstal R.M., Holl R.W., Fleming G.A., Heinemann L. (2017). Improving the clinical value and utility of CGM systems: Issues and recommendations: A joint statement of the European Association for the Study of Diabetes and the American Diabetes Association Diabetes Technology Working Group. Diabetologia.

[4] Fokkert M.J., van Dijk P.R., Edens M.A., Abbes S., de Jong D., Slingerland R.J., Bilo H.J. (2017). Performance of the FreeStyle Libre Flash glucose monitoring system in patients with type 1 and 2 diabetes mellitus. BMJ Open Diabetes Res. Care.

[5] Gubala V., Harris L.F., Ricco A.J., Tan M.X., Williams D.E. (2012). Point of care diagnostics: Status and future. Anal. Chem..

[6] Wiig H., Swartz M.A. (2012). Interstitial fluid and lymph formation and transport: Physiological regulation and roles in inflammation and cancer. Physiol. Rev.

[7] Paliwal S., Hwang B.H., Tsai K.Y., Mitragotri S. (2013). Diagnostic opportunities based on skin biomarkers. Eur. J. Pharm. Sci..

[8] Samant P.P., Prausnitz M.R. (2018). Mechanisms of sampling interstitial fluid from skin using a microneedle patch. Proc. Natl. Acad. Sci. USA.

[9] Lundberg J., Rudling M., Angelin B. (2013). Interstitial fluid lipoproteins. Curr. Opin. Lipidol..

[10] Vaught J.B., Henderson M.K. (2011). Biological sample collection, processing, storage and information management. IARC Sci. Publ..

[11] Cahill E.M., Keaveney S., Stuettgen V., Eberts P., Ramos-Luna P., Zhang N., Dangol M., O’Cearbhaill E.D. (2018). Metallic microneedles with interconnected porosity: A scalable platform for biosensing and drug delivery. Acta Biomater..

[12] Fu Y., Zhao J., Dong Y., Wang X. (2020). Dry Electrodes for Human Bioelectrical Signal Monitoring. Sensors.

[13] Ren L., Xu S., Gao J., Lin Z., Chen Z., Liu B., Liang L., Jiang L. (2018). Fabrication of Flexible Microneedle Array Electrodes for Wearable Bio-Signal Recording. Sensors.

[14] Chien M.-N., Fan S.-H., Huang C.-H., Wu C.-C., Huang J.-T. (2022). Continuous Lactate Monitoring System Based on Percutaneous Microneedle Array. Sensors.

[15] Chang H., Zheng M., Yu X., Than A., Seeni R.Z., Kang R., Tian J., Khanh D.P., Liu L., Chen P. (2017). A Swellable Microneedle Patch to Rapidly Extract Skin Interstitial Fluid for Timely Metabolic Analysis. Adv. Mater..

[16] Caffarel-Salvador E., Brady A.J., Eltayib E., Meng T., Alonso-Vicente A., Gonzalez-Vazquez P., Torrisi B.M., Vicente-Perez E.M., Mooney K., Jones D.S. (2015). Hydrogel-forming microneedle arrays allow detection of drugs and glucose in vivo: Potential for use in diagnosis and therapeutic drug monitoring. PLoS ONE.

[17] Takeuchi K., Takama N., Kinoshita R., Okitsu T., Kim B. (2020). Flexible and porous microneedles of PDMS for continuous glucose monitoring. Biomed. Microdevices.

[18] Ye R., Yang J., Li Y., Zheng Y., Yang J., Li Y., Liu B., Jiang L. (2020). Fabrication of Tip-Hollow and Tip-Dissolvable Microneedle Arrays for Transdermal Drug Delivery. ACS Biomater. Sci. Eng..

[19] Bolton C.J.W., Howells O., Blayney G.J., Eng P.F., Birchall J.C., Gualeni B., Roberts K., Ashraf H., Guy O.J. (2020). Hollow silicon microneedle fabrication using advanced plasma etch technologies for applications in transdermal drug delivery. Lab Chip.

[20] Nicholas D., Logan K.A., Sheng Y., Gao J., Farrell S., Dixon D., Callan B., McHale A.P., Callan J.F. (2018). Rapid paper based colorimetric detection of glucose using a hollow microneedle device. Int. J. Pharm..

[21] Larraneta E., Lutton R.E., Woolfson A.D., Donnelly R.F. (2016). Microneedle arrays as transdermal and intradermal drug delivery systems: Materials science, manufacture and commercial development. Mater. Sci. Eng. R Rep..

[22] Khanna P., Luongo K., Strom J.A., Bhansali S. (2010). Axial and shear fracture strength evaluation of silicon microneedles. Microsyst. Technol..

[23] Onishi J., Makabe K., Matsumoto Y. (2008). Fabrication of micro sloping structures of SU-8 by substrate penetration lithography. Microsyst. Technol..

[24] Tan J.Y., Kim A., Kim J.J.K. Fabrication and Characterization of Hollow Microneedle Array Using Diffraction UV Lithography. Proceedings of the 2021 21st International Conference on Solid-State Sensors, Actuators and Microsystems (Transducers).

[25] Lim J., Tahk D., Yu J., Min D.H., Jeon N.L. (2018). Design rules for a tunable merged-tip microneedle. Microsyst. Nanoeng..

[26] Bae W.-G., Ko H., So J.-Y., Yi H., Lee C.-H., Lee D.-H., Ahn Y., Lee S.-H., Lee K., Jun J. (2019). Snake fang–inspired stamping patch for transdermal delivery of liquid formulations. Sci. Transl. Med..

[27] Kochhar J.S., Soon W.J., Choi J., Zou S., Kang L. (2013). Effect of microneedle geometry and supporting substrate on microneedle array penetration into skin. J. Pharm. Sci..

[28] Park H.-H., Seong M., Sun K., Ko H., Kim S.M., Jeong H.E. (2017). Flexible and shape-reconfigurable hydrogel interlocking adhesives for high adhesion in wet environments based on anisotropic swelling of hydrogel microstructures. ACS Macro Lett..

[29] Xu Q., McMichael P., Creagh-Flynn J., Zhou D., Gao Y., Li X., Wang X., Wang W. (2018). Double-cross-linked hydrogel strengthened by UV irradiation from a hyperbranched PEG-based trifunctional polymer. ACS Macro Lett..

[30] Martinez A.W., Phillips S.T., Butte M.J., Whitesides G.M. (2007). Patterned paper as a platform for inexpensive, low-volume, portable bioassays. Angew. Chem..

[31] Songjaroen T., Dungchai W., Chailapakul O., Henry C.S., Laiwattanapaisal W. (2012). Blood separation on microfluidic paper-based analytical devices. Lab Chip.

[32] Fu E., Ramsey S.A., Kauffman P., Lutz B., Yager P. (2011). Transport in two-dimensional paper networks. Microfluid. Nanofluid..

[33] Tahk D., Paik S.-M., Lim J., Bang S., Oh S., Ryu H., Jeon N.L. (2017). Rapid large area fabrication of multiscale through-hole membranes. Lab Chip.

[34] Lee H., Bonfante G., Sasaki Y., Takama N., Minami T., Kim B. (2020). Porous microneedles on a paper for screening test of prediabetes. Med. Devices Sens..

[35] Takahashi H., Kan T., Lee G., Dönmez N., Kim J., Park J., Kim D., Heo Y.J. (2020). Self-focusing 3D lithography with varying refractive index polyethylene glycol diacrylate. Appl. Phys. Express.

