# Supplementary material for: Hollow Microneedles on a Paper Fabricated by Standard Photolithography for the Screening Test of Prediabetes
Source: Sensors (Basel). 2022 Jun 2;22(11):4253. doi: 10.3390/s22114253 (PMC9185271; doi:10.3390/s22114253)
Supplement: Supplementary file 1 [file sensors-22-04253-s001.zip › Supplementary Information.pdf]

Supplementary Information

Hollow Microneedles on a Paper Fabricated by Standard Photo-lithography for the Screening Test of Prediabetes

Tianwei Wu [1], Xueqiu You [1,2,*] and Zhong Chen [1,*]

[1] Department of Electronic Science, Fujian Provincial Key Laboratory of Plasma and Magnetic Resonance, State Key Laboratory of Physical Chemistry of Solid Surfaces, Xiamen University, Xiamen 361005, China; 34320191150168@stu.xmu.edu.cn
[2] School of Information Engineering, Jimei University, Xiamen 361021, China
* Correspondence: youxueqiuqqq@126.com (X.Y.); chenz@xmu.edu.cn (Z.C.)

As shown in Figure S1(a-c), when the offset distance ranges from 0 μm to 30 μm, the opening profile also increases. The microneedle heights for different UV intensity is shown in Figure S1(d).

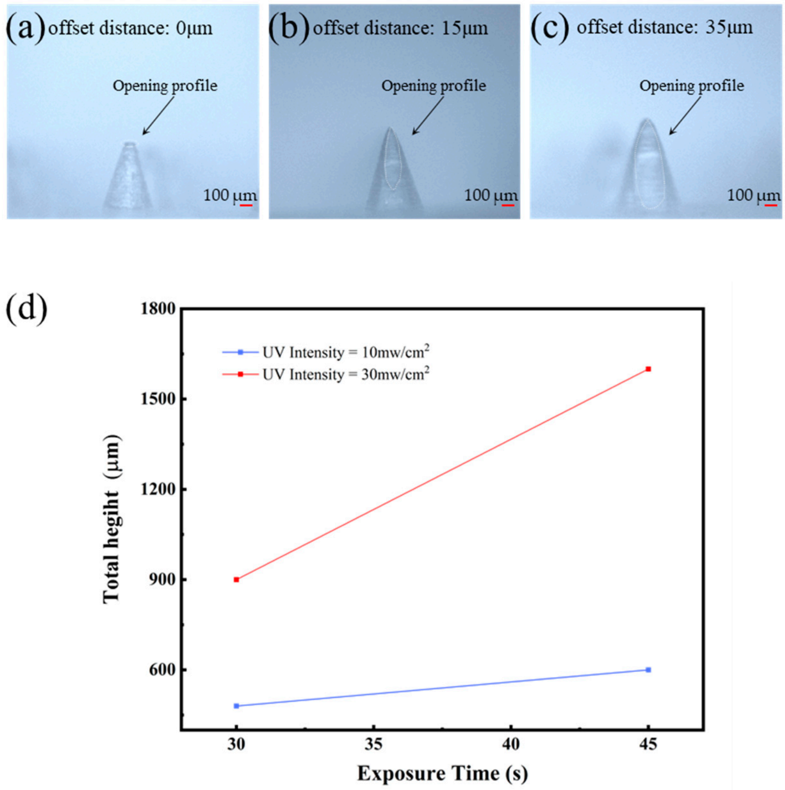

Figure S1. (a-c) Optical images of different offset distances. (d) The microneedle heights for different UV intensity.

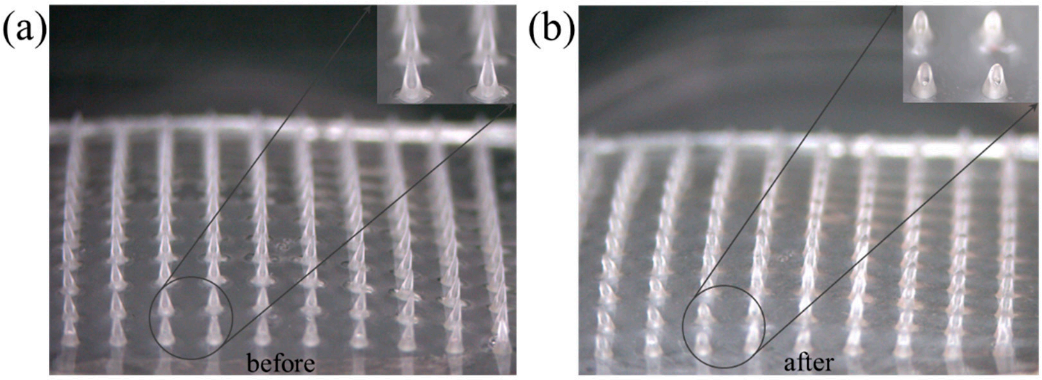

Figure S2. The optical image before (a) and after (b) the penetration.
