# Supplementary figures and images for: Hollow Microneedles on a Paper Fabricated by Standard Photolithography for the Screening Test of Prediabetes
Source: Sensors (Basel). 2022 Jun 2;22(11):4253. doi: 10.3390/s22114253 (PMC9185271; doi:10.3390/s22114253)

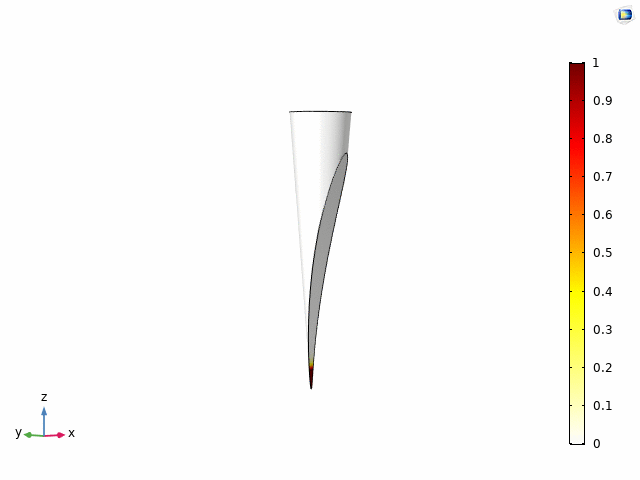

Supplement: Supplementary file 1 [file sensors-22-04253-s001.zip › movie S1.gif]
